# Therapeutic potential of vitamin D in improving antioxidant defense and blood rheology in a rat model of experimental diabetes mellitus

**DOI:** 10.1515/med-2026-1403

**Published:** 2026-03-12

**Authors:** Nino Sakhanberidze, Manana Namoradze, Nino Charkviani, Maia Mantskava, Nana Momtselidze, Davit Delibashvili, Natia Gamkrelidze

**Affiliations:** Department of Pathophysiology and Endocrinology, Tbilisi State Medical University, Tbilisi, Georgia; Department of Pathophysiology, Tbilisi State Medical University, Tbilisi, Georgia; Department of Endocrinology, Tbilisi State Medical University, Tbilisi, Georgia; Department of Physics, Biophysics, Biomechanics and Informational Technologies, Tbilisi State Medical University, Tbilisi, Georgia; Department of Rheological and Diagnostic-Analytical Services, Ivane Beritashvili Experimental Center of Biomedicine, Tbilisi, Georgia

**Keywords:** vitamin D, experimental diabetes mellitus, streptozotocin (STZ), oxidative stress, antioxidant defense, blood rheology

## Abstract

**Objectives:**

To evaluate the preventive and corrective effects of vitamin D on antioxidant defense and blood rheology in rats with streptozotocin (STZ)-induced diabetes mellitus.

**Methods:**

Forty male rats (10 weeks old, ∼200 g) were randomly assigned to four groups: Group I – Intact controls; Group II – Diabetic rats (STZ only); Group III – Preventive (vitamin D for 14 days before STZ and continued throughout the experiment); Group IV – Therapeutic (vitamin D from day 3 post-STZ). DM was induced with streptozotocin (30 mg/kg), and vitamin D was administered orally at 300 IU/day, according to a previous dose-control experiment. Data from the 21st experimental day were evaluated. Blood samples collected at this point were used to determine catalase (CAT), superoxide dismutase (SOD), and blood rheological parameters.

**Results:**

Vitamin D supplementation enhanced antioxidant-related parameters and improved blood rheological indices, particularly when administered therapeutically.

**Conclusions:**

Vitamin D shows therapeutic potential in attenuating oxidative imbalance and improving blood rheology in streptozotocin-induced diabetes mellitus.

## Introduction

Diabetes mellitus is a complex metabolic disorder characterized by persistent hyperglycemia resulting from absolute insulin deficiency and/or impaired insulin action, which disrupts normal glucose utilization [1]. In addition to impaired glucose uptake, increased endogenous glucose production through enhanced gluconeogenesis and glycogenolysis further contributes to sustained hyperglycemia. Globally, an estimated 589 million adults (aged 20–79 years) – approximately one in nine individuals – are currently living with diabetes, a figure projected to rise to 853 million by 2050 [2]. Diabetes mellitus includes several clinical types, such as type 1 diabetes, type 2 diabetes, gestational diabetes, and less common forms like latent autoimmune diabetes in adults (LADA), maturity-onset diabetes of the young (MODY), and secondary diabetes due to pancreatic disease or drug use [3]. Despite their diverse etiologies and clinical courses, all forms share chronic hyperglycemia resulting from impaired carbohydrate metabolism.

Among the mechanisms implicated in the progression of diabetes mellitus, oxidative stress has emerged as a central pathological factor. Chronic hyperglycemia promotes excessive generation of reactive oxygen species (ROS) through multiple hyperglycemia-associated biochemical pathways [4], [5], [6], [7], [8], [9]. These mechanisms contribute to the development of diabetic microvascular and macrovascular complications, such as nephropathy, neuropathy, retinopathy, and cardiovascular disease [6], [8], [9], [10], [11].

Sustained hyperglycemia is associated with increased oxidative imbalance and lipid peroxidation, which contribute to tissue injury and vascular dysfunction in diabetes mellitus [5], [12], [13], [14], [15], [16]. Consequently, targeting oxidative stress has emerged as a relevant therapeutic strategy, with antioxidant-based approaches reported to improve oxidative stress markers and metabolic profiles in diabetes mellitus [17], 18]. Oxidative stress–induced lipid peroxidation represents a hallmark of diabetes mellitus and contributes to impaired antioxidant defenses, microcirculatory dysfunction, and hemodynamic disturbances [19]. In this context, altered blood rheology is closely associated with oxidative stress, as ROS-mediated damage to erythrocyte membranes and hemoglobin oxidation impair red blood cell deformability and flow properties [20]. Moreover, oxidative stress promotes platelet aggregation, endothelial dysfunction, and inflammatory responses, thereby exacerbating vascular complications and highlighting the importance of timely intervention [21], 22].

Given these insights, vitamin D has emerged as a promising endogenous regulator of redox homeostasis and metabolic function. Beyond its classical role in calcium–phosphate balance, vitamin D has been reported to influence insulin signaling and glucose metabolism, and vitamin D deficiency has been associated with hyperglycemia as well as both microvascular and macrovascular complications of diabetes mellitus [4], [5], [6], [8], [23], [24], [25]. These effects are mediated through mechanisms involved in metabolic regulation and cellular homeostasis [26], [27], [28], [29], [30].

Beyond its metabolic actions, vitamin D may also influence vascular health and diabetes mellitus–related vascular dysfunction. Collectively, these effects provide a rationale for investigating vitamin D as a modulator of oxidative stress and blood rheological disturbances in diabetes mellitus. Of particular interest, alterations in blood rheology have been increasingly recognized as important contributors to vascular complications in diabetes mellitus. Multiple studies have documented significant changes in blood viscosity, erythrocyte deformability, and microcirculatory flow during the diabetic state [31], [32], [33]. Both clinical and experimental evidence consistently demonstrate biomechanical dysfunction of red blood cells, particularly in type 2 diabetes mellitus, where such alterations exacerbate vascular injury and metabolic stress [34], 35]. Furthermore, oxidative and inflammatory processes act synergistically to aggravate these rheological disturbances, thereby amplifying the risk of diabetic vascular complications [36]. Although these alterations are more pronounced in type 2 diabetes mellitus, blood rheological impairments are evident across different forms of diabetes [32], [34], [35], [36], [37], [38], [39].

It remains unclear whether vitamin D deficiency serves as a cause or a consequence of diabetes mellitus and its complications. Moreover, the precise role of vitamin D in modulating blood rheology, particularly under varying shear conditions, has not been well defined. The global rise in diabetes mellitus prevalence, the absence of effective preventive strategies, and inconsistent findings regarding viscosity-related changes underscore the need for novel investigative approaches.

We selected the streptozotocin (STZ)-induced diabetes mellitus model due to its selective toxicity toward pancreatic β-cells and its ability to induce diabetes in a rapid and reproducible manner. While multiple experimental approaches exist to mimic specific types of diabetes mellitus, the STZ model does not fully replicate the complex pathogenesis of human diabetes. Nevertheless, it is widely recognized as a scientifically valid and reliable model for studying fundamental mechanisms of diabetes mellitus, such as hyperglycemia and oxidative stress. In addition, we evaluated several induction methods beforehand and identified STZ as the most suitable approach for rheology assessment [40].

Considering the pivotal role of oxidative stress in the development of diabetes-associated vascular dysfunction and the accumulating evidence supporting a role for vitamin D in redox regulation and blood rheology control, the present study was undertaken to examine the effects of vitamin D on antioxidant defense systems and blood rheology in experimental diabetes mellitus. We hypothesized that vitamin D supplementation would enhance antioxidant enzyme activity and ameliorate diabetes mellitus-related alterations in blood rheological parameters, with outcomes dependent on the timing of intervention. Accordingly, the research question addressed whether preventive and therapeutic vitamin D administration differentially modulate oxidative stress markers and blood rheological properties in a streptozotocin-induced diabetes mellitus model.

## Materials and methods

### Experimental design and animal treatment

An experiment was conducted on 40 male non-breed-specific rats. All animals were male, as male rats are more sensitive to streptozotocin (STZ) than females, and to avoid potential confounding effects related to hormonal fluctuations. The age was 10 weeks and the weight 200 g. They were fed with a diet specifically formulated for laboratory rats. Environmental conditions: 24 °C±1 °C, 55 % ± 5 % humidity, 12-h light-dark cycle. All procedures were performed according to established guidelines [38].

**Figure 1:**
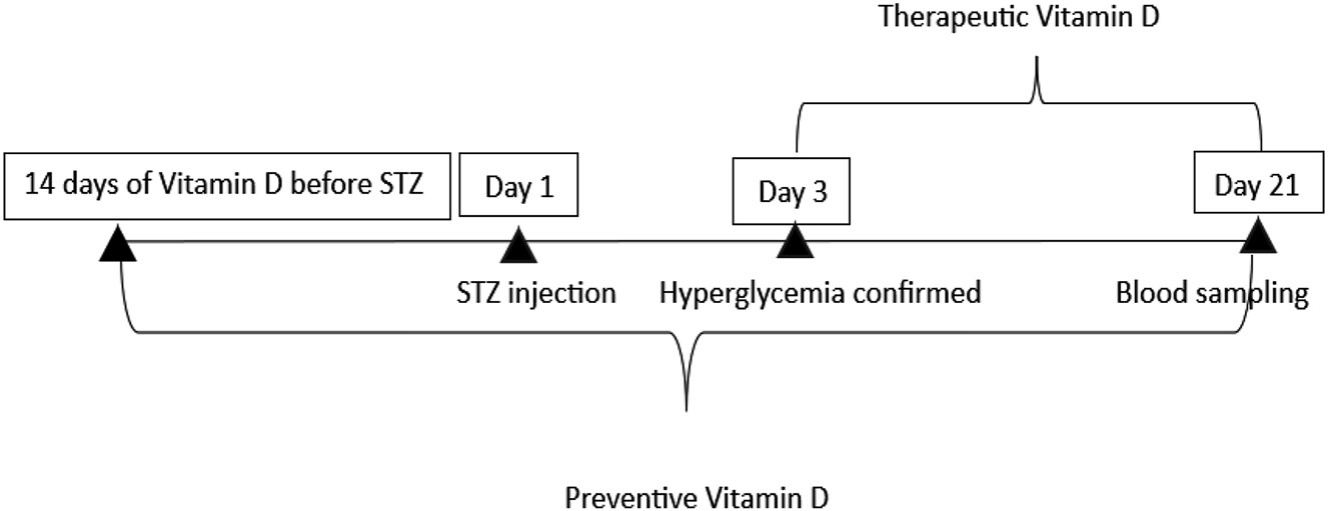
Experimental timeline of preventive and therapeutic vitamin D administration in streptozotocin (STZ)-induced diabetes mellitus.

The animals were allocated into four experimental groups: the control group, consisting of intact healthy rats; the diabetic group, comprising rats intraperitoneally injected with STZ to induce diabetes mellitus without vitamin D supplementation; the preventive group, in which rats received vitamin D supplementation for 14 days prior to STZ administration and throughout the experimental period; and the therapeutic group, in which vitamin D supplementation was initiated on day 3 post-STZ injection and maintained until the end of the study (Table 1, Figure 1).

**Table 1:** Study design and experimental groups.

No.	Group	Description	Number
1	Control group	Consists of healthy rats	10
2	STZ control group	STZ-induced diabetes mellitus, no vitamin D	10
3	Preventive STZ + Vit D	vitamin D (300 IU/day, oral) for 14 days before STZ and continued until day 21	10
4	Therapeutic STZ + Vit D	vitamin D (300 IU/day, oral) from day 3 post-STZ until day 21	10

The selected dose was based on a preliminary dose–response experiment in which several daily doses of vitamin D (30, 150, 200, 300, 400, 600, and 800 IU/day) were evaluated to minimize the risk of hypercalcemia while identifying an effective therapeutic range. Higher doses (400–800 IU/day) showed a tendency toward increased serum calcium levels that remained within the physiological range but approached the upper limit of normal. The 300 IU/day dose demonstrated a favorable balance, maintaining calcium levels within the normal range without approaching the upper threshold. Therefore, 300 IU/day was selected as the safest and most appropriate dose for the present study.

The duration of the study was 21 days following the induction of experimental diabetes. On Day 1, diabetes mellitus was induced by a single streptozotocin (STZ) injection. Prior to STZ administration, body mass and fasting blood glucose levels of all rats were recorded. Hyperglycemia was confirmed on Day 3, indicating successful establishment of diabetes mellitus.

To mimic the natural route of intake, vitamin D was administered orally to the rats each day. Body weight and blood glucose levels were measured on days 1, 3, and 21. In addition, glucose levels were monitored between these time points using a glucometer to evaluate the stability of the diabetic state. Blood samples were collected after an 18-h fasting period, and vitamin D administration was discontinued one day prior to blood collection.

### Diabetes induction

The injection solution was prepared in accordance with established guidelines [41]. A streptozotocin (STZ) dose of 30 mg/kg was selected, as this concentration is associated with a low mortality rate (approximately one rat per 10). Each rat received a single intraperitoneal (IP) injection of STZ, dissolved in freshly prepared sodium citrate buffer (50 mM, pH 4.5). Given the high sensitivity of STZ to environmental factors, solutions were prepared immediately before administration and maintained under appropriate temperature conditions. The control group received an intraperitoneal injection of sodium citrate buffer (pH 4.5) without STZ. To ensure stability and efficacy, injections were completed within 10 min of preparation. All rats were fasted prior to STZ administration. Following the STZ injection, animals received 10 % sucrose solution. Diabetes induction was confirmed on the third day post-injection by measuring blood glucose levels using a glucometer. In all STZ-injected rats, hyperglycemia exceeding 19 mmol/L and polyuria were consistently observed on the third day, serving as definitive indicators of diabetes mellitus development.

Preventive vitamin D was administered for 14 days prior to STZ injection and continued until day 21. Therapeutic vitamin D was initiated on day 3 after confirmation of hyperglycemia and continued until day 21. Vitamin D was administered orally at a dose of 300 IU/day. Blood sampling was performed on day 21.

### Glucose monitoring

Glucose levels were monitored throughout the experiment using a Contour Plus glucometer (Ascensia Diabetes Care, Basel, Switzerland). Blood samples were obtained via tail vein puncture to assess and confirm the stability of the diabetic state in the experimental groups.

### Blood biochemical analysis

At the end of the experimental period, serum glucose concentration was determined by the glucose oxidase–peroxidase (GOD-POD) enzymatic colorimetric method (BIO-LABO, France) using a semi-automated biochemical analyzer (URIT-880, China). Serum levels of catalase and superoxide dismutase were measured using FineTest ELISA kits (Fine Biotech Co., Ltd., China), reported in mIU/mL and ng/mL, respectively. All procedures were carried out according to the manufacturers’ instructions.

### Blood rheology

RBC aggregation index represents aggregated RBC’ area ratio against the whole area of RBCs. RBC aggregation was evaluated with the recently developed “Georgian technique” providing us with direct and quantitative data. Blood samples were centrifuged and about 0.1 mL blood was diluted 1:200 in their own plasma in the Thoma pipettes preliminary rinsed with 5 % sodium citrate solution without the addition of any other anticoagulants to the blood under study. Following standard mixing the diluted blood was placed into a glass chamber 0.1 mm high. The quantitative index of RBC aggregation, which was assessed with a special program at the Texture Analysis System (TAS-plus, “Leitz, Germany), represented the ratio between aggregated and non-aggregated erythrocytes.

Blood plasma viscosity was examined in the capillary viscometer at 37 °C. Diameter of the capillary was about 1.8 mm. Displacement of plasma samples was induced by the gravity force related to the difference of niveaux of the plasma under study - about 65 (without application of additional pressure). For evaluation of the plasma viscosity in centipoises (cP), we determined the calibration factor (F). Blood plasma viscosity was calculated by multiplying the time for plasma displacement through the capillary by the instrument calibration factor.

Erythrocyte deformability index (EDI) was performed with the aid of the nucleopore membrane filter method, which is based on assessing the velocity of the erythrocytes passage through the very small pores (5 µm, which is a diameter of the smallest capillary) of the filter, at constant pressure (10 cm of water column) and temperature (37 °C). Obtaining the pure RBC was performed by centrifuging the blood sample at 3,000 rpm, for 15 min. The resulting plasma was aspirated with a micropipette and the remaining blood cells were added with bovine serum albumin (0.2 mg per 5 mL) dissolved in the phosphate buffer. Then the blood was centrifuged a second time at 1,000 rpm for 5 min. The precipitated erythrocytes, as well as a thin layer of leukocytes and thrombocytes, were separated from the phosphate buffer. This procedure was repeated three times. Purified RBC mass was diluted in the phosphate buffer, with a hematocrit of 10 %. Evaluation of the deformability index implied measuring a velocity of the erythrocyte passage through the filter (mm/min) was recorded. The high-quality polycarbonate filters (with 5 µm diameter pores) were used in measuring procedures (modification of filtration methods).

### Body weight measurement

The initial body mass of each rat was recorded prior to the start of the experiment. Additionally, body weights for all groups were measured on day three. All measurements were recorded in grams using a digital scale with a precision of 0.1 g. These weight measurements were necessary for adjusting the streptozotocin (STZ) dosing and for assessing general health status and weight patterns.

### Statistical analysis

Statistical analyses were performed using Origin 8.1 software. Data normality was assessed using the Shapiro–Wilk test prior to parametric analysis. Differences among experimental groups were analyzed using one-way analysis of variance (ANOVA). When the ANOVA indicated statistically significant differences, post-hoc pairwise comparisons were conducted using Student’s t-tests. To account for multiple comparisons and prevent Type I error inflation, a Bonferroni correction was applied, and the significance threshold was adjusted accordingly (p<0.05/n). Results are presented as mean±standard deviation (SD). Exact p-values are provided in the figure legends and/or Results section where applicable. A p-value < 0.05 was considered statistically significant.

### Ethical approval

All animal experiments were conducted in accordance with institutional regulations and internationally accepted guidelines for the care and use of laboratory animals. The experimental protocol was reviewed and approved by the Ethics Committee of Tbilisi State Medical University (Protocol No. 60–1002, approved on 10 February 2023). All procedures were designed to minimize animal pain and distress and were performed in compliance with the principles of the 3Rs (Replacement, Reduction, and Refinement).

### Research ethics

All animal experiments were conducted in accordance with institutional regulations and internationally accepted guidelines for the care and use of laboratory animals. The experimental protocol was reviewed and approved by the Ethics Committee of Tbilisi State Medical University (Protocol No. 60-1002; approved on 10 February 2023). All procedures were performed in compliance with the principles of the 3Rs (Replacement, Reduction, and Refinement) and were designed to minimize animal pain and distress.

### Informed consent

Not applicable.

## Results

### Body mass changes

**Figure 2:**
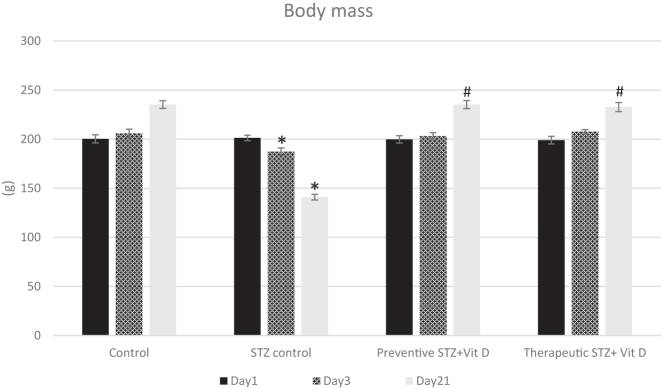
Body mass of experimental groups on days 1, 3, and 21. Data are presented as mean±SD. “*” - p<0.05 compared with the control group at the same time point; “#” - p<0.05 compared with the STZ control group at the same time point.

Body mass was assessed on days 1 (baseline, prior to STZ administration), 3, and 21 of the experimental period. Diabetes mellitus was confirmed on day 3 in STZ-injected rats (STZ control, preventive, and therapeutic) by the presence of marked hyperglycemia (>19 mmol/L) accompanied by polyuria. By day 21, the STZ control group exhibited a significant reduction in body mass compared with the healthy control group (p<0.05). In contrast, administration of vitamin D in both preventive and therapeutic groups was associated with higher body mass values. At day 21, body mass in the vitamin D–treated groups was significantly higher than in the STZ control group (p<0.05) and tended towards control values (Figure 2).

### Glycemic levels

**Figure 3:**
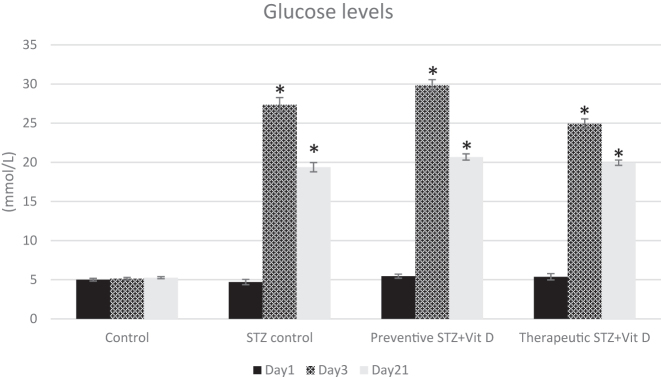
Fasting blood glucose levels (mmol/L) measured in the experimental groups on days 1, 3, and 21. Data are presented as mean±SD. “*” - p<0.05 compared with the control group at the same time point; No statistically significant differences in fasting blood glucose levels were observed between STZ control and vitamin D–treated groups at any time point (p>0.05).

Blood glucose concentrations were measured on days 1 (baseline), 3, and 21 of the experiment. On day 3 following streptozotocin administration, all STZ-injected groups (STZ control, preventive, and therapeutic) exhibited marked hyperglycemia, with glucose levels exceeding 19 mmol/L, confirming the successful induction of diabetes mellitus (p<0.05). Elevated glucose levels were maintained throughout the experimental period and persisted through day 21 in all STZ-injected groups. Preventive and therapeutic vitamin D administration did not result in significant changes in blood glucose concentrations compared with the STZ control group at any time point examined (p>0.05) (Figure 3).

### Antioxidant defense parameters (catalase and superoxide dismutase)

**Figure 4:**
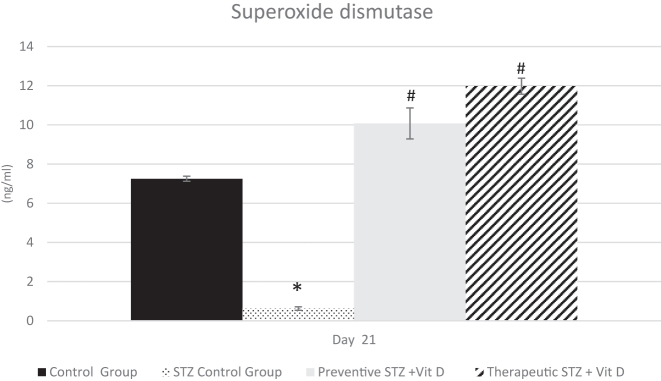
Superoxide dismutase (SOD) level (ng/mL) in all experimental groups on day 21. Data are presented as mean±SD. “_*_” - p<0.05 compared with the control group at the same time point; “_#_” - p<0.05 compared with the STZ control group at the same time point.

**Figure 5:**
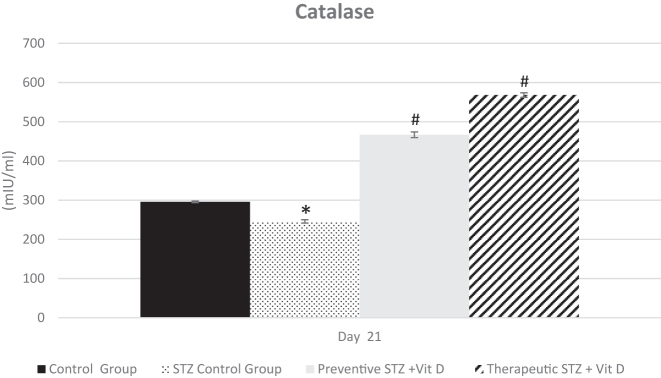
Catalase (CAT) activity (mIU/mL) measured in all experimental groups on day 21. Data are presented as mean±SD. “_*_” - p<0.05 compared with the control group at the same time point; “_#_” - p<0.05 compared with the STZ control group at the same time point.

On day 21 of the experiment, marked changes in antioxidant defense parameters were observed among the experimental groups. The STZ control group exhibited a significant reduction in catalase (CAT) and superoxide dismutase (SOD) compared with the normal control group (p<0.05). Preventive vitamin D administration significantly increased CAT and SOD relative to the STZ control group (p<0.05). Similarly, therapeutic vitamin D treatment resulted in a significant elevation of both antioxidant enzymes compared with the diabetic group (p<0.05). Overall, vitamin D treatment, whether preventive or therapeutic, resulted in a marked increase in antioxidant defense parameters relative to the STZ control group (Figures 4 and 5).

### Blood rheology

Blood rheological parameters, including the erythrocyte aggregation index (EAI), erythrocyte deformability index (EDI), and plasma viscosity (PIV), were evaluated in all experimental groups on day 21. Compared with the control group, the STZ control group (group 2) exhibited increased erythrocyte aggregation index (≈20 %) and plasma viscosity (≈19 %), accompanied by a marked reduction in erythrocyte deformability index (≈17–18 %) (p<0.05). In the preventive vitamin D group (group 3), EAI and PIV remained elevated and EDI remained reduced, with no significant differences compared with the STZ control group (p>0.05), indicating a lack of preventive effect. In contrast, therapeutic vitamin D administration significantly reduced erythrocyte aggregation index and plasma viscosity and significantly improved erythrocyte deformability index relative to the STZ control group (p<0.05), partially restoring these parameters toward control values. Collectively, these findings indicate that improvements in blood rheological parameters were evident primarily following therapeutic vitamin D administration (Table 2).

**Table 2:** Erythrocyte aggregation index (EAI), erythrocyte deformability index (EDI), and plasma viscosity (PIV) in all experimental groups on day 21.

Group	EAI, %	EDI, mm/min	PIV (cP)
1. Control Group	15.1±0.14	2.40±0.08	1.30±0.10
2. STZ Control Group	18.1±0.20^a^	1.98±0.10^a^	1.55±0.10^a^
3. Preventive STZ + Vit D	17.0±0.10	2.00±0.08	1.50±0.10
4. Therapeutic STZ + Vit D	15.0±0.18^b^	2.30±0.12^b^	1.30±0.10^b^

Data are presented as mean±SD. “^a^” - p<0.05 compared with the control group at the same time point; “^b^” - p<0.05 compared with the STZ control group at the same time point.

## Discussion

In this experimental study, we found that vitamin D exerted both preventive and therapeutic effects on antioxidant defense systems, while its effects on blood rheological parameters were primarily therapeutic, in streptozotocin-induced diabetes mellitus. Specifically, vitamin D administration led to an increase in antioxidant defense parameters, including catalase and superoxide dismutase, in both preventive and therapeutic contexts. In addition, blood rheological parameters were improved only in the therapeutic group, indicating that vitamin D may contribute to correcting blood flow alterations associated with diabetes mellitus. These findings suggest that therapeutic vitamin D administration may modulate antioxidant defense systems and improve blood rheological properties, supporting its potential role in the management of diabetes-related complications.

Our findings are consistent with previous studies demonstrating that vitamin D supplementation enhances the activity of key antioxidant enzymes, including superoxide dismutase and catalase, thereby mitigating diabetes mellitus–associated oxidative injury [42], 43]. However, the distinct effects of preventive vs. therapeutic vitamin D administration on antioxidant status and blood rheological parameters remain insufficiently explored. In line with earlier reports, vitamin D supplementation in the present study did not produce significant changes in glucose levels [44], 45]. Notably, despite the absence of glucose lowering, marked improvements in erythrocyte aggregation, plasma viscosity, and erythrocyte deformability were observed in the therapeutic group. This pattern suggests that the beneficial effects of vitamin D on blood rheology may be mediated, at least in part, through mechanisms independent of glycemic control. As discussed previously, oxidative stress represents a central pathogenetic mechanism in diabetes mellitus and its vascular complications. Chronic hyperglycemia drives excessive reactive oxygen species generation, mitochondrial dysfunction, protein glycation, and activation of alternative metabolic pathways, culminating in persistent redox imbalance and endothelial injury. These processes promote vascular remodeling, inflammation, and microcirculatory dysfunction. Importantly, oxidative stress is tightly linked to disturbances in blood rheological properties, including increased plasma viscosity, enhanced erythrocyte aggregation, and reduced erythrocyte deformability. Such alterations have been reported to further exacerbate tissue hypoxia and sustain oxidative injury, thereby establishing a self-perpetuating pathological cycle [37], [38], [39, 46]. Consistent with this concept, accumulating evidence indicates that blood rheology is strongly influenced by oxidative stress, lipid peroxidation, and structural alterations of the erythrocyte membrane – processes that are not solely dependent on hyperglycemia [47], 48]. Oxidative damage to erythrocyte membranes has been shown to impair deformability and promote aggregation, underscoring the critical role of oxidative and inflammatory conditions in determining red blood cell mechanical properties. Vitamin D deficiency has been associated with increased oxidative stress and diminished antioxidant capacity, whereas supplementation has been shown to attenuate reactive oxygen species production, modulate inflammatory responses, and support endothelial stability [49]. Notably, these effects may occur without consistent changes in glycemic indices such as HbA1c or fasting glucose [50], 51]. Taken together, these glucose-independent actions support the concept that vitamin D may influence blood rheology through redox- and endothelial-related pathways rather than direct glucose lowering. These findings point to a potential therapeutic window that represents a key scientific interest and justifies the rationale of the present investigation.

In the literature, several mechanisms have been proposed to explain the antioxidant and vascular effects of vitamin D, including modulation of Nrf2-related signaling, VDR–RXR–mediated gene regulation, mitochondrial function, and anti-inflammatory pathways. However, these mechanisms were not directly examined in the present study and should therefore be regarded as plausible explanations rather than definitive conclusions [52], 53].

The present findings indicate that the physiological and pathophysiological state at the time of supplementation may influence the blood rheological response to vitamin D. Although antioxidant defense increased in both preventive and therapeutic groups, improvements in blood rheological parameters were observed only when vitamin D was administered after the establishment of hyperglycemia-associated oxidative stress. This suggests that vitamin D supplementation during a phase of active metabolic and vascular disturbance may coincide with a therapeutic window in which its effects on blood rheology become more apparent. It is also important to note that changes in blood rheological properties likely depend on time-dependent processes, such as erythrocyte membrane remodeling and endothelial recovery. While the precise molecular pathways remain to be clarified, these observations underscore the importance of timing and context in vitamin D administration and highlight the need for future studies incorporating molecular and endothelial markers to better define the mechanisms involved.

Our study demonstrates that vitamin D may serve as a promising therapeutic agent for managing oxidative stress and improving blood rheological parameters in diabetes mellitus. These findings underscore the importance of considering not only dosage but also the timing of supplementation to maximize its potential benefits. Nevertheless, further investigations are required to validate these effects and to establish optimal strategies for vitamin D use in clinical practice.

Several aspects of the present study should be considered. First, it was conducted in an experimental model of streptozotocin-induced diabetes mellitus, which is specifically used to evaluate the effects of hyperglycemia but may not fully capture the complex pathophysiology of human diabetes. Second, the study was conducted over a 21-day experimental period, which was sufficient to evaluate the effects of vitamin D supplementation on oxidative stress and blood rheological parameters within this time frame; however, the duration does not extend to later-stage outcomes related to the progression of diabetes-related complications. Although a preliminary dose-control experiment was performed in our laboratory to identify a safe and effective dosage – during which several daily doses of vitamin D (30–800 IU/day) were evaluated to avoid the risk of hypercalcemia – the main study implemented only a single dose (300 IU/day). As a result, potential dose–dependent relationships could not be assessed, and future studies incorporating a dose–response design will be necessary to determine the optimal therapeutic range. Although additional metabolic and systemic markers, including insulin, HbA1c, lipid profile, calcium–phosphate balance, and inflammatory mediators, may provide complementary insights, the present study was intentionally focused on oxidative status and blood rheological parameters.

## Conclusions

In conclusion, this study demonstrates that vitamin D exerts preventive and corrective effects on antioxidant defense systems in streptozotocin (STZ)-induced diabetes mellitus, whereas its beneficial impact on blood rheological parameters is evident primarily following therapeutic administration. These findings highlight the importance of supplementation timing and support the potential role of vitamin D as an adjunctive approach for mitigating oxidative stress and improving blood rheology in diabetes mellitus. Further studies investigating molecular mechanisms and long-term outcomes are required to confirm and extend these findings.
